# Flow Injection Analysis with Electrochemical Detection for Rapid Identification of Platinum-Based Cytostatics and Platinum Chlorides in Water

**DOI:** 10.3390/ijerph110201715

**Published:** 2014-02-04

**Authors:** Marketa Kominkova, Zbynek Heger, Ondrej Zitka, Jindrich Kynicky, Miroslav Pohanka, Miroslava Beklova, Vojtech Adam, Rene Kizek

**Affiliations:** 1 Department of Chemistry and Biochemistry, Faculty of Agronomy, Mendel University in Brno, Zemedelska 1, CZ-613 00 Brno, Czech Republic; E-Mails: kominkova.marketa@gmail.com (M.K.); heger@mendelu.cz (Z.H.); zitkao@seznam.cz (O.Z.); zitkao@seznam.cz (V.A.); 2 Department of Ecology and Diseases of Game, Fish and Bees, Faculty of Veterinary Hygiene and Ecology, University of Veterinary and Pharmaceutical Sciences, Palackeho 1–3, CZ-612 42 Brno, Czech Republic; E-Mail: beklovam@vfu.cz; 3 Central European Institute of Technology, Brno University of Technology, Technicka 3058/10, CZ-616 00 Brno, Czech Republic; E-Mail: miroslav.pohanka@gmail.com; 4 Department of Geology and Pedology, Faculty of Forestry and Wood Technology, Mendel University in Brno, Zemedelska 1, CZ-613 00, Czech Republic; E-Mail: jindrich.kynicky@mendelu.cz; 5 Karel Englis College, Sujanovo nam, 356/1, CZ-602 00 Brno, Czech Republic

**Keywords:** platinum-based cytostatics, platinum chlorides, flow injection analysis with electrochemical detection, hydrodynamic voltammograms

## Abstract

Platinum-based cytostatics, such as cisplatin, carboplatin or oxaliplatin are widely used agents in the treatment of various types of tumors. Large amounts of these drugs are excreted through the urine of patients into wastewaters in unmetabolised forms. This phenomenon leads to increased amounts of platinum ions in the water environment. The impacts of these pollutants on the water ecosystem are not sufficiently investigated as well as their content in water sources. In order to facilitate the detection of various types of platinum, we have developed a new, rapid, screening flow injection analysis method with electrochemical detection (FIA-ED). Our method, based on monitoring of the changes in electrochemical behavior of analytes, maintained by various pH buffers (Britton-Robinson and phosphate buffer) and potential changes (1,000, 1,100 and 1,200 mV) offers rapid and cheap selective determination of platinum-based cytostatics and platinum chlorides, which can also be present as contaminants in water environments.

## 1. Introduction

The platinum group elements (PGEs)—platinum, palladium, rhodium, ruthenium, and the relatively rare iridium and osmium—are among the less abundant elements in the environment. Their extreme resistance to other chemicals and great mechanical properties make them ideally suited for a large number of applications. In particular, platinum has been found to be very useful in a broad range of sectors [1]. Platinum metals are used in jewelry and in catalytic converters in the automotive industry, and currently also in medicine, where this metal is increasingly being used as material for production of stents, spinal fixations, hip or knee implants and as a component of antineoplastic agents a [2]. Due to the steady increase in their use, PGEs are also potential environmental contaminants, which tend to bioaccumulate in various plant and animal tissues representing a serious threat to organisms [3]. The largest toxicological problems are caused by platinum chlorides and coordination complexes used in chemotherapy [4,5,6,7,8,9]. 

Due to their negative effects on organisms it is necessary to properly identify the presence of PGEs and especially cytostatics in the water environment which serves as distribution route [10]. The most widely used platinum-based cytostatic, cisplatin, is applied in concentrations of 75–100 mg.m^−2^ of body surface area, oxaliplatin in concentrations of 150 mg.m^−2^ and carboplatin in concentrations of 400 mg.m^−2^. Some 75% of the applied amounts may be excreted through urine into wastewaters [11]. These values indicate the potential seriousness of wastewater contamination with platinum-based cytostatics and highlight the importance of determination of their content. For this purpose a wide range of methods, such as atomic absorption spectrometry (AAS), the most commonly applied analytical method for PGE detection, inductively coupled plasma (ICP), optical emission spectrometry (OES) or mass spectrometry (MS) can be utilized [12,13]. Liquid chromatography (LC) in tandem with mass spectrometry can also be applied for the determination of platinum-based cytostatics [14]. Electrochemical methods based on the catalytic properties of specific platinum compounds are characterized by high sensitivity to the presence of other platinum based compounds [15,16,17]. All of these methods require various sample pre-treatments, long analysis times and expensive instrumentation. 

It is important to explore new ways and methods to simplify, accelerate and reduce the costs of PGE analyses. One possibility is offered by flow injection analysis with electrochemical detection (FIA-ED), based on which now we suggest a procedure providing rapid detection of PGEs and recognition of the presence of platinum-based cytostatics in contaminated wastewaters.

## 2. Experimental Section

### 2.1. Chemicals and pH Measurement

Standards of PtCl_2_, PtCl_4_, RhCl_3_ and PdCl_2_ were obtained from Sigma-Aldrich (St. Louis, MO, USA). Oxaliplatin was purchased from Merck & Co (Whitehouse Station, NJ, USA), carboplatin was obtained from Teva UK (Castleford, UK), and cisplatin was from EBEWE Pharma (Unterach am Attersee, Austria). Other chemicals were purchased from Sigma-Aldrich in ACS purity unless noted otherwise. Stock standard solutions of platinum species (1 mg·mL^−1^) were prepared in ACS water with 1% HCl (*v/v*) added to the increase solubility of the metal ions. Working standard solutions of the analyzed platinum species were prepared daily immediately prior to the use by the dilution of the stock solutions to the final concentration of 10 µg·mL^−1^. All solutions were prepared in deionized water obtained by the use of Aqual 25 reverse osmosis equipment (Aqual s.r.o., Brno, Czech Republic). Deionized water was further purified by using a Direct-Q 3 UV Water Purification System apparatus equipped with a UV lamp (Millipore, Billerica, MA, USA). The output resistance was set to 18 MΩ∙cm^−1^. The value of pH was measured using a WTW inoLab pH meter (Weilheim, Germany) equipped with a terminal Level 3, controlled by the WTW MultiLab Pilot software.

### 2.2. River Water Sample

A sample of river water (Ponavka stream from the city of Brno, Czech Republic) was filtered through a 0.45 μm nylon filter disc (Millipore) prior to FIA-ED analysis.

### 2.3. FIA-ED System

The instrument for flow injection analysis with electrochemical detection (FIA-ED) consisted of a solvent delivery pump operating within the range of 0.001–9.999 mL.min^−1^ (Model 582 ESA Inc., Chelmsford, MA, USA) and an electrochemical detector. The electrochemical detector includes a low-volume flow-through analytical cell (Model 5040, ESA), which consists of a glassy carbon electrode as a working electrode, a hydrogen-palladium electrode as a reference electrode and an auxiliary electrode, and a Coulochem III unit as a control potentiostat module. The sample (20 μL) was injected using an autosampler (Model 542, ESA). Buffers with different pH, chosen for the ability to maintain constant conditions (pH) during the measurements (phosphate buffer with pH 5.5, 6.5, 7.5, and Britton-Robinson buffer with pH 2, 3, 3.5, 4, 5 and 6, respectively) were used as mobile phases (for optimized conditions see the Results and Discussion section). Detection was carried out at different potentials (100, 200, 300, 400, 500, 600, 700, 800, 900, 1,000, 1,100, and 1,200 mV) which were applied to obtain hydrodynamic voltammograms (HDVs) of the individual platinum species. The data obtained were analyzed with the Clarity software (Version 1.2.4, Data Apex, Prague, Czech Republic). The experiments were carried out at a temperature of 25 °C. A glassy carbon electrode was polished mechanically with alumina (0.1 μm, ESA Inc.) and sonicated at room temperature for 5 min using a Sonorex Digital 10 P Sonicator (Bandelin, Berlin, Germany) at 40 W [18]. 

### 2.4. Descriptive Statistics

Mathematical analysis of the experimental data and their graphical interpretation were carried out by the Microsoft Office tools (MS Excel^®^, MS Word^®^, and MS PowerPoint^®^). All results were expressed as mean ± standard deviation (SD) unless noted otherwise. The detection limits (3 × signal/noise, S/N) were calculated according to Long and Winefordner [19], where N was expressed as a standard deviation of noise determined in the signal domain unless stated otherwise.

## 3. Results and Discussion

**Figure 1:**
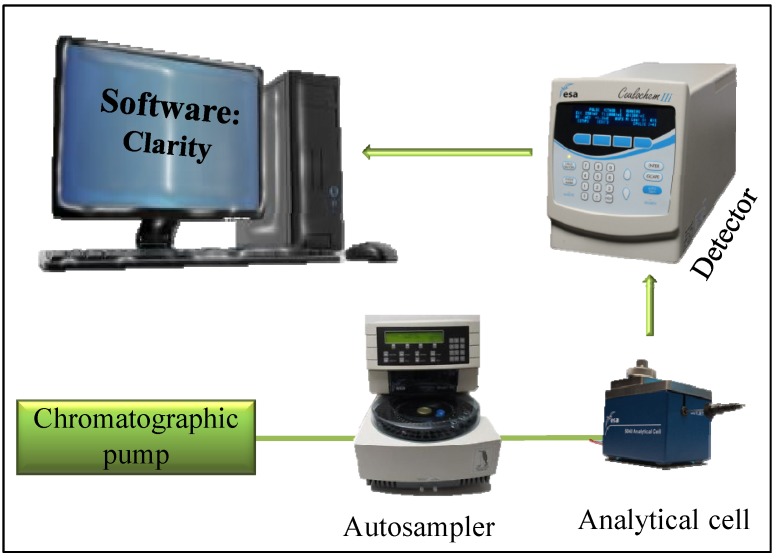
Scheme of FIA-ED system used for determination of platinum-based cytostatics and platinum chlorides.

Widely used platinum-based cytostatics are released in unmetabolised forms through the urine of patients and thus they may be found in large amounts in hospital wastewaters [20]. The presence of these pollutants can be proved using a variety of analytical methods, usually requiring large operational costs and complicated sample pre-treatments. Flow injection analysis (FIA), allowing automation and high throughput analysis, is not characterized by these issues and can be easily used for rapid detection of various types of analytes. FIA is based on the injection of a acurate volume of the sample into a flowing mobile phase and its subsequent physical and chemical transformation, which allows the use of different types of detectors [21,22]. A typical FIA configuration consists of a pump, an injection valve, a flow-through detector, and a signal output device. Our FIA configuration, shown in Figure 1, utilized electrochemical detection, particularly amperometric detection using a working glassy carbon electrode (GCE). Similar configurations were used for detection of thiol compounds and for studying their interaction with cisplatin [18].

**Figure 2:**
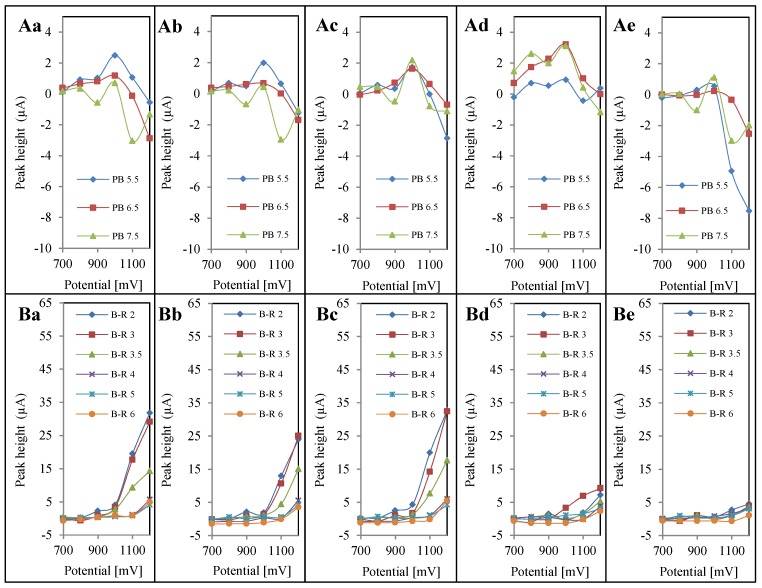
Hydrodynamic voltammograms (HDVs). All HDVs were measured with concentration of 10 µg.mL^−1^ of PGEs using (**A**) phosphate buffer (PB) for (**Aa**) oxaliplatin; (**Ab**) cisplatin; (**Ac**), carboplatin; (**Ad**) PtCl_2_; (**Ae**) PtCl_4_; and (**B**) Britton-Robinson buffer (B-R) for (**Ba**) oxaliplatin; (**Bb**) cisplatin; (**Bc**) carboplatin; (**Bd**) PtCl_2_; (**Be**) PtCl_4_.

Besides, the response of the analytes depends on the pH of the mobile phase and the potential applied for the electrochemical detection. Conditions for the detection of oxaliplatin, cisplatin, carboplatin, PtCl_2_ and PtCl_4_, (in concentrations of 10 μg∙mL^−1^) were evaluated using Britton-Robinson buffer at pH 2, 3, 3.5, 4, 5 and 6, and phosphate buffer at pH 5.5, 6.5 and 7.5. For individual analytes, including the blank sample (detector response after injection of the solution used for sample dilution), hydrodynamic voltammograms (HDVs) were constructed showing the electrochemical behavior of each analyte in all the mentioned buffers. For accurate recognition of qualitative changes of analyte behavior during the electrochemical measurements, subtraction of a blank HDV from each analytes’ HDV was carried out. This step was performed in order to improve the selectivity of detection, and to eliminate false positive results. The resulting curves can be seen in Figure 2. 

**Figure 3:**
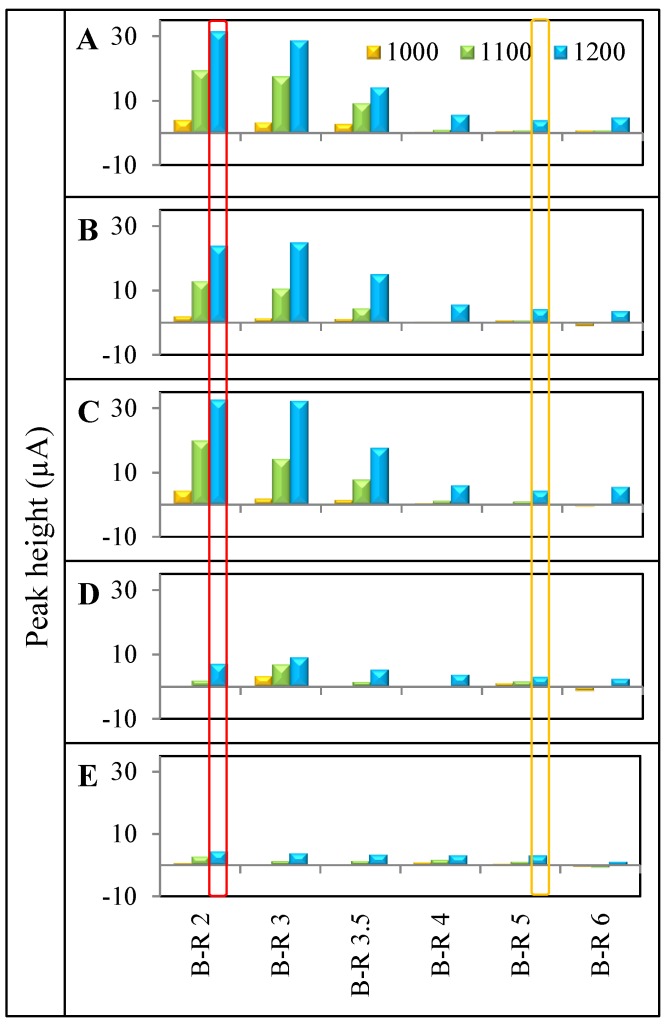
Expression of ideal potentials (range 1000–1200 mV) in buffers Britton-Robinson buffer (B-R) with pH 2–6 for each of PGEs obtained from HDVs. All maximums were carried out with PGEs concentration of 10 µg.mL^−1^. Ideal potentials for (**A**) oxaliplatin; (**B**) cisplatin; (**C**) carboplatin; (**D**) PtCl_2_; (**E**) PtCl_4_ are shown. Optimal potential and pH buffer for determination of platinum-based cytostatics from platinum chlorides is highlighted with red color. Potential and pH buffer, characterizing concentration of platinum in sample is highlighted with orange.

According to these measurements, three potentials were selected (1000, 1100 and 1200 mV) as the ones with the highest detector responses. Signal values are shown in Figure 3 for better clarity. On the basis of these obtained values, an overview of the different electrochemical behavior of platinum-based cytostatics and platinum chlorides was obtained. Inclusion of this procedure eliminates irregularities caused by the presence of signal when analyzing solutions in the absence of an analyte, because the solvent used for sample dissolution causes physical and chemical changes on the electrode surface, and thereby also a detector response. In Figure 2(Aa–Ae), the HDVs of analytes after subtraction of blank solution in the environment, maintained by phosphate buffer, can be seen. It clearly follows from the results obtained that analytes did not show linearity and produced significantly lower signals when compared to values measured in the presence of Britton-Robinson buffer (Figure 2(Ba–Be)). Therefore, it appeared that phosphate buffer is not suitable for the detection of the studied platinum compounds. When comparing the values of the analyte signals measured in the presence of Britton-Robinson buffer with potentials of 1000, 1100, and 1200 mV (Figure 3) noticeable differences between detection of platinum-based cytostatics and platinum chlorides can be observed.

This distinction is most obvious when using Britton-Robinson buffer of pH 2 and an applied potential of 1200 mV (Figure 3, highlighted with red rectangle). On the other hand when using a Britton-Robinson buffer of pH 5 and an applied potential of 1200 mV (Figure 3 highlighted with an orange rectangle), the signals of all the tested platinum compounds only showed negligible differences. Under the conditions maintained by pH 5 Britton-Robinson buffer, the detector response reached 24–32 µA for platinum-based cytostatics (31–32 µA for oxaliplatin and carboplatin and 24 µA for cisplatin), while the platinum chlorides’ detector responses reached currents within a range from 4.5 to 7.2 µA. In contrast, the utilization of Britton-Robinson buffer of pH 5 showed partial compensation of all platinum compounds currents (4.1–4.2 µA for platinum-based cytostatics and 3.1 µA for platinum chlorides). 

**Figure 4:**
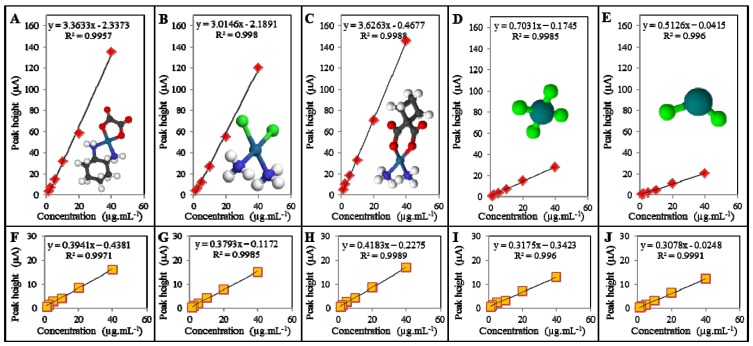
Calibration curves for individual platinum analytes measured at applied potential of 1200 mV, using Britton-Robinson buffer at pH 2: (**A**) oxaliplatin; (**B**) cisplatin; (**C**) carboplatin; (**D**) PtCl_2_; (**E**) PtCl_4_ and using Britton-Robinson buffer at pH 5: (**F**) oxaliplatin; (**G**) cisplatin; (**H**) carboplatin; (**I**) PtCl_2_; (**J**) PtCl_4_.

Differing signal strength of platinum-based cytostatics and platinum chlorides, determined using FIA-ED, was confirmed also by the calibration curves of the individual platinum compounds measured in the presence of Britton-Robinson buffer of pH 2 (Figure 4(A–E)) and pH 5 (Figure 4(F–J)), where the formulas of the individual tested platinum compounds can be seen too. Besides the confirmation of lower signal provided by platinum chlorides (approximately 4–7 times) compared to platinum-based cytostatics, and there was also a slight decrease of the cisplatin signal versus the signals of carboplatin and oxaliplatin. This phenomenon can be explained by the presence of chlorine in the cisplatin molecule, nevertheless impact on the recognition between the presences of platinum-based cytostatics and platinum chlorides was not affected significantly by the presence of chlorine in the cisplatin structure.

From the calibration curves, the mechanism of the method that could serve as a tool for distinguishing of platinum-based cytostatics from platinum chlorides can be derived. In the case of the positive signal seen when using Britton-Robinson buffer of pH 2 and the subsequent positive signal when using the same buffer of pH 5, another evaluation follows. If the peak height of platinum measured in Britton-Robinson of pH 2 shows an approximately 1.5–2.5 × higher value that in the same buffer with pH 5, the result points to platinum chlorides. In the case that the peak height of platinum analyzed in the Britton-Robinson buffer of pH 2 is 5–9 × higher than in the same buffer of pH 5, platinum drugs are present in the water sample. Our method is based only on changing two buffers forming the mobile phases of the FIA-ED system. This approach is rapid, cheap and it can be miniaturized and thus used for biosensor applications. The analysis cost ranges from approximately 1–2 Euros, whereas the instrumentation cost includes only a low-pressure pump, an injection valve and an electrochemical detector.

**Figure 5:**
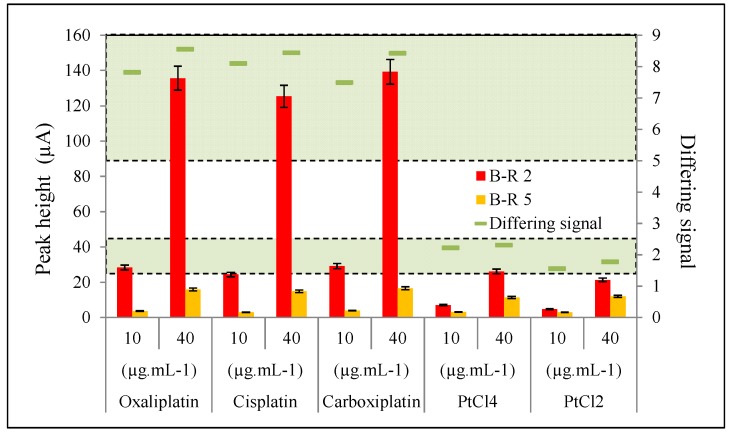
Results of analyzes of water from the stream after the addition of each variant of platinum. Samples in the B-R buffer, pH 2 are shown in a red column, and pH 5 as an orange column. Green marks show the ratio of buffers used, and concurrently also determine the variations of platinum.

The functionality of this method was verified by analyzing a sample of water from the Ponavka stream. Platinum cytostatics and platinum chloride were added to this water in concentrations of 10 and 40 μg∙mL^−1^. The samples were analyzed and calculated using the proposed method shown above. The data obtained from the analysis are shown in Figure 5. The accompanying results show that the proposed method is useful for analyzing water samples contaminated with platinum species.

## 4. Conclusions

Based on the obtained results, we suggest a rapid and inexpensive method with the potential to be miniaturized. The FIA-ED method, based on the comparison of the responses of an amperometric detector using a glassy carbon working electrode, can be used to distinguish the presence of platinum-based cytostatics from platinum chlorides. This method could be applicable for hospital wastewater evaluation or for rapid, screening analyses in places where the manipulation with these cytostatics is performed and there is an increased threat of environment contamination. 
